# A widely adaptable approach to generate integration-free iPSCs from non-invasively acquired human somatic cells

**DOI:** 10.1007/s13238-014-0117-1

**Published:** 2014-11-22

**Authors:** Zhichao Ding, Lina Sui, Ruotong Ren, Yanjun Liu, Xiuling Xu, Lina Fu, Ruijun Bai, Tingting Yuan, Ying Hao, Weiqi Zhang, Huize Pan, Wensu Liu, Han Yu, Concepcion Rodriguez Esteban, Xiaobing Yu, Ze Yang, Jian Li, Xiaomin Wang, Juan Carlos Izpisua Belmonte, Guang-Hui Liu, Fei Yi, Jing Qu

**Affiliations:** 1 National Laboratory of Biomacromolecules, Institute of Biophysics, University of the Chinese Academy of Sciences, Chinese Academy of Sciences, Beijing, 100101 China; 2 Department of Endocrinology, 306th Hospital of PLA, Beijing, 100101 China; 3 Science and Development, Xtem, Foshan, 528000 China; 4 State Key Laboratory of Reproductive Biology, Institute of Zoology, Chinese Academy of Sciences, Beijing, 100101 China; 5 Beijing Hospital of the Ministry of Health, Beijing, 100073 China; 6 Beijing Institute for Brain Disorders, Beijing, 100069 China; 7 Gene Expression Laboratory, Salk Institute for Biological Studies, 10010 North Torrey Pines Road, La Jolla, CA 92037 USA; 8 Center for Molecular and Translational Medicine, Beijing, 100101 China; 9 Department of Molecular and Cellular Physiology, Stanford University School of Medicine, Stanford, CA 94305 USA

**Dear Editor**,

Human pluripotent stem cells including human embryonic stem cells (ESCs) and induced pluripotent stem cells (iPSCs) are cells displaying abilities of unlimited self-renewal and differentiation into any somatic cell type. These unique properties make them increasingly attractive for novel applications in disease modeling, drug discovery, and cell therapy (Buganim et al., 2014; Liu et al., 2011; Liu et al., 2012; Sanchez Alvarado and Yamanaka, 2014). Moreover, iPSCs hold great potential for personalized cell therapy as they avoid some of the ethical concerns as well as the immunological rejection issues ascribed to ESCs. To date, iPSCs can be generated from various cell sources that range from commonly used skin fibroblasts to rarely employed blood cells, mostly through virus-based reprogramming methods with different efficiencies (Patel and Yang, 2010). However, the isolation of these original human cells often involves an invasive sample acquisition procedure. Therefore, human somatic cells that can be conveniently obtained in a non-invasive manner, including human endometrium cells (EMCs) and human urine-derived cells (UCs), may represent more convenient and promising sources for the generation of iPSCs. This is especially crucial for patients hypersensitive to any invasive operation (i.e. patients with Diabetes mellitus (DM) and patients with hemophilia). On the other hand, non-integrative reprogramming methods including those mediated by episomal vectors (Okita et al., 2011), Sendai virus vectors (Seki et al., 2012) and small chemicals (Hou et al., 2013; Lin et al., 2009), are increasingly regarded as superior alternatives to viral approaches. Recently, an elegant study has demonstrated that iPSCs could be established by an episomal system comprised of miR-302–367 from human UCs (Xue et al., 2013). Here we report on the generation of integration-free human endometrium-derived iPSCs (emiPSCs) and urine-derived iPSCs (uiPSCs) by a modified episomal reprogramming system. Subsequently, we show that these iPSCs can robustly differentiate into several valuable lineage-specific cell types, including pancreatic progenitor cells (PPs) and neural stem cells (NSCs).

Healthy and patient donors at an age ranging from 8 to 83 were recruited with signed and approved consents by our institutional committees. 1–3 mL menstrual blood or 200–500 mL urine was centrifuged and washed by phosphate-buffered saline (PBS) containing Penicillin-Streptomycin, and then cultured in mesenchymal cell medium and renal epithelial cell medium respectively for 1–2 weeks. In total, we established 5 EMC lines from healthy donors and 16 UC lines from healthy individuals and type II DM patients. These primary EMCs and UCs proliferate well and could be passaged for at least 6 passages in culture. Next, two EMC lines and two UC lines from healthy individuals and one UC line from a DM patient were randomly chosen and subjected to cellular reprogramming. A normal fibroblast line isolated from a 9-year old donor (Liu et al., 2011) was used as the reprogramming control. Here, we utilized a modified system by which integration-free iPSCs have been successfully generated from fibroblasts of a *FANCA* mutant Faconi anemia (FA) patient (Liu et al., 2014; Seki et al., 2012). This approach was proven to be robust as FA patient fibroblasts were reported to be refractory to somatic cell reprogramming (Liu et al., 2014; Raya et al., 2009). We also proved that cells reprogrammed through this strategy maintained genomic integrity even in a compromised disease context, as FA patient cells have a defective DNA repair system (Liu et al., 2014; Raya et al., 2009). To initiate cell reprogramming, we electroporated a cocktail of episomal vectors that encode for human OCT4, SOX2, KLF4, LIN28, L-MYC, and a p53-specific shRNA, into EMCs and UCs respectively. Sodium butyrate was included in the medium to facilitate cell reprogramming-associated epigenetic remodeling (Liu et al., 2014; Mali et al., 2010). After seeding on top of mouse embryonic fibroblast (MEF) feeders, small iPSC-like colonies appeared around 10 days after electroporation for all selected lines. Typical iPSC colonies with human ESC morphology were ready for picking and expanding at around day 20 (Fig. 1A). All resulting iPSC lines demonstrated typical ESC features, including a high nucleus/cytoplasma ratio, positive alkaline phosphatase (AP) activity, expression of pluripotency markers OCT4, SOX2, and NANOG, as well as demethylation of the *OCT4* promoter (Fig. 1A–D). These cells were stably cultured on MEF or Matrigel for more than 20 passages, without acquiring any karyotypic aberrance (Fig. 1E). Additionally, quantitative RT-PCR (RT-qPCR) analysis showed no residual episomal vector element in the established iPSC lines (Fig. 1F).

**Figure 1:**
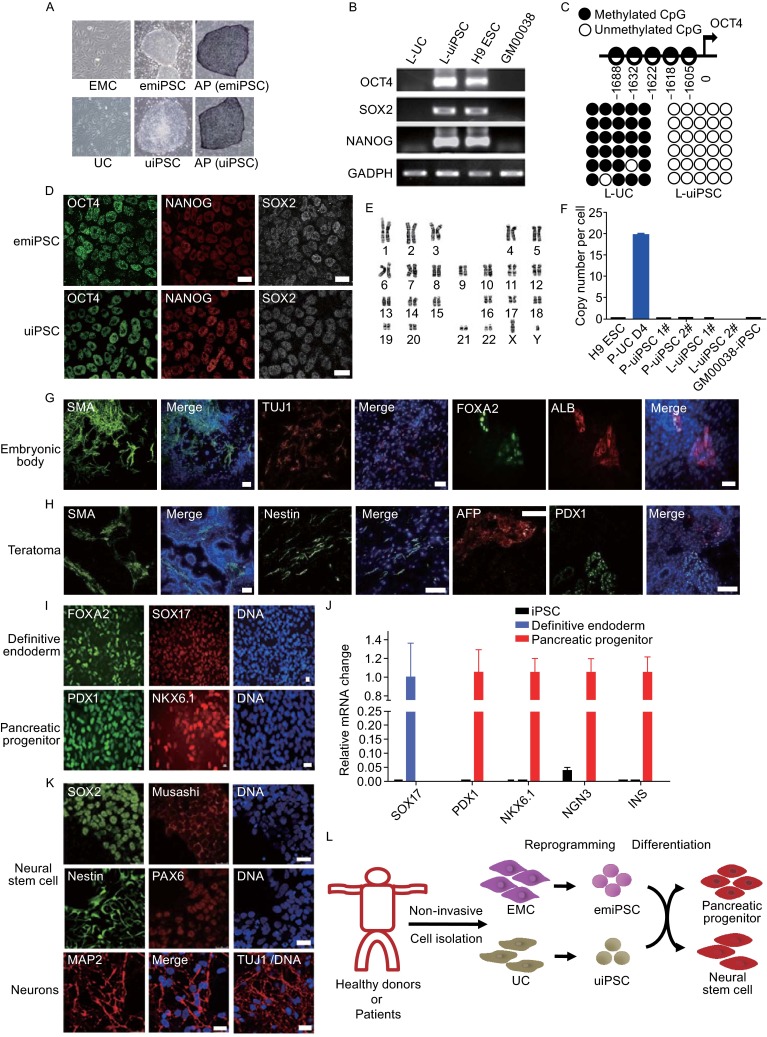
**Establishment and characterization of emiPSCs and uiPSCs**. (A) Cellular morphology and AP staining of emiPSCs and uiPSCs generated from human EMCs and UCs. (B) RT-qPCR analysis of endogenous pluripotency gene expression in indicated lines. H9 human ESCs and GM00038 human fibroblasts were used as positive and negative controls, respectively. (C) DNA methylation analysis of the *OCT4* promoter in the L-UC and L-uiPSC lines. The positions of the CpG dinucleotides relevant with the *OCT4* transcription start site are present. (D) Immunofluorescence analysis of pluripotency markers OCT4, NANOG, and SOX2 in L-uiPSCs. Nuclear DNA was stained with Hoechst 33258. (E) G-band analysis of L-uiPSCs showing a normal karyotype. (F) Copy number quantification of the episomal vector element EBNA1 in indicated iPSC lines. H9 human ESCs were employed as a negative control, and the P-UCs 4 days (P-UC D4) after electroporation with the episomal reprograming factors were used as a positive control. (G–H) Spontaneous EB differentiation *in vitro* (G) and teratoma analysis *in vivo* (H) verify the differentiation potentials of iPSCs towards endodermal, mesodermal, and ectodermal tissues. Scale bar, 75 μm for the teratoma analysis. (I–J) Immunofluorescence analysis (I) and RT-qPCR (J) demonstrate the up-regulation of FOXA2, SOX17 in DE; and up-regulation of PDX1, NKX6.1, NGN3, and INS in PPs. (K) Immunofluorescence analysis shows NSCs generated from iPSCs express specific makers SOX2, Musashi, Nestin, and PAX6; and neurons generated from iPSCs express neuronal makers MAP2 and TUJ1. All scale bars represent 25 μm unless otherwise specified. (L) Schematic presentation of establishing integration-free emiPSCs and uiPSCs and generating their PP and NSC derivatives *in vitro*

We further examined the *in vitro* and *in vivo* differentiation potentials of the generated iPSCs. The *in vitro* spontaneous differentiation potential of iPSCs was investigated by embryoid body (EB)-based differentiation assays. As shown in Fig. 1G, iPSCs effectively differentiated into TUJ1 (ectoderm), SMA (mesoderm), as well as FOXA2 and ALB (endoderm)-positive cells upon spontaneous differentiation. Furthermore, after injection into immune-deficient NOD/SCID mice, iPSCs were able to develop into teratomas composed of cells from all three germ-layers marked by SMA, AFP, PDX1, and Nestin expressions (Fig. 1H).

Next, we investigated the potency of the iPSCs in directed cell differentiations. PPs and NSCs were chosen because of their value in potential applications. In order to generate PPs, iPSCs were firstly treated with a combination of activin A and Wnt3a to initiate the definitive endoderm cell (DE) differentiation, a step resulting in 95% endodermal progenitors co-expressing FOXA2 and SOX17 (Fig. 1I and 1J). After treating the endodermal progenitors with Retinoic acid, FGF10, Noggin, and Cyclopamine-KAAD, we obtained a highly pure population of PPs positive for the pancreatic progenitor marker PDX1 (Fig. 1I and 1J). As anticipated, these PPs also expressed NKX6.1 and NGN3. An upregulation of insulin gene (INS) expression at transcriptional level was also observed (Fig. 1I and 1J). Additionally, we differentiated iPSCs toward NSCs by treatment with a chemically defined medium comprising of hLIF, SB431542, CHIR99021, Compound E, and dorsomorphin (Liu et al., 2012). These resulting NSCs expressed typical neural progenitor markers SOX2, PAX6, Musashi, and Nestin (Fig. 1K). As a characteristic feature of neural progenitor cells, these cells were able to efficiently differentiate into MAP2 and TUJ1-positive neurons when exposed to a differentiation medium containing ascorbic acid, dbcAMP, BDNF and GDNF for 14 days (Fig. 1K).

In summary, we demonstrated here that integration-free iPSCs can be efficiently and consistently obtained from non-invasively acquired human EMCs and UCs, even in a defective disease context (i.e. type II DM) (Fig. 1L). Given the fact that a similar strategy has been proven to be effective in reprograming diseased somatic cells inherently resistant to the regular cell reprogramming procedure (Liu et al., 2014), we speculate that this methodology could be universally adaptable for various applications. The autologous PP and NSC derivatives generated from emiPSCs and uiPSCs could be further subjected to animal transplantation studies (Sui et al., 2013; Zhang et al., 2009), and even potentially be applied to human regenerative medicine settings. The robustness and non-invasive nature of our system to generate iPSCs from human samples may enormously fuel the application of human iPSCs towards novel cell therapy as well as applications in disease modeling and drug discovery in the near future.

## Electronic supplementary material

Below is the link to the electronic supplementary material.

Supplementary material 1 (PDF 292 kb)
