# Supplementary material for: A widely adaptable approach to generate integration-free iPSCs from non-invasively acquired human somatic cells
Source: Protein Cell. 2014 Nov 22;6(5):386–9. doi: 10.1007/s13238-014-0117-1 (PMC4417681; doi:10.1007/s13238-014-0117-1)
Supplement: Supplementary file 1 — Supplementary material 1 (PDF 292 kb) [file 13238_2014_117_MOESM1_ESM.pdf]

Supplementary methods

Plasmids and antibodies

Episomal plasmids including pCXLE-hOCT3/4-shp53-F (27077), pCXLE-hUL (27080) and pCXLE-hSK (27078) were all purchased from Addgene (Okita et al., 2011). Antibodies used were purchased from different companies indicated as below: anti-OCT-3/4 (sc-5279) and anti- SOX2 (sc-17320) were from Santa Cruz Biotechnology; anti-NANOG (ab21624) and anti-Musashi (ab52865) from Abcam; anti-SMA(A5228), anti-TUJ1(T2200) and anti-AFP(A8452) from Sigma; anti-SOX17(MAB1924) and anti-PDX1(AF2419) from R&D system; anti-FOXA2 (8186) from Cell Signaling Technology; anti-Nestin (MAB5326) from Millipore; anti-ALB (CL2513A) from Cedarlane labs; anti-PAX6 (PRB-278P) from Covance; anti-NKX6.1(F65A10) from Developmental Studies Hybridoma Bank; all the secondary antibodies from Invitrogen.

Ethical statement and establishment of EMCs and UCs

The animal experiments and human subject researches were ethically approved by Animal Care and Use Committee at IBP, and the ethics committee of 306 hospital of PLA. All the donors of human EMCs and UCs have signed informed consent document. Human EMCs and UCs were collected and washed by phosphate-buffered saline (PBS) with Penicillin-Streptomycin (Gibco), and then cultured in mesenchymal cell medium (90% MEMα (Gibco), 10% Fetal bovine serum (FBS) (Gibco), 1 ng/ml FGF2 (The joint Protein Central), and 1% Penicillin-Streptomycin (Gibco)), and renal epithelial cell medium (90% Dulbecco's Modified Eagle's Medium/F12 (DMEM/F12) (Gibco) and 10% FBS (Gibco) with Renal Cell Growth Medium (REGM) SignleQuot Kit supplemets (LONZA)) as described previously (Zhou et al., 2012), respectively.

iPSC generation

For iPSC generation, the reprogramming was performed as described previously with small modifications (Liu et al., 2014; Okita et al., 2011). Briefly, one million human EMCs and UCs were nucleofected with episomal plasmids pCXLE-hOCT3/4-shp53-F, pCXLE-hUL and pCXLE-hSK. The electroporated EMCs and UCs were re-seeded onto Mitomycin C (biomol international) inactivated mouse embryonic fibroblast (MEF) feeders in human ESC medium with addition of 500 μM sodium butyrate (Sigma) after four days. After additional 20-30 days, the iPSC colonies were mechanically passaged and expanded onto new MEF feeders in human ESC medium. Human fibroblasts (GM00038) were cultured in high glucose DMEM (Gibco) medium with 10% FBS (Gibco) as reported (Liu et al., 2011), before the reprogramming.

Nucleic acid analysis

Total RNA was extracted by TRIzol (Invitrogen). The first cDNA synthesis and quantitative RT–PCR were carried out with Universal RiboClone cDNA Synthesis System (Promega) and SYBR Green PCR Master Mix (Applied Biosystems), respectively. The copy number of transgenes in established iPSCs was determined as reported (Okita et al., 2011). The primers of pluripotency genes for semi-quantification PCR were reported previously (Okita et al., 2011). The other qPCR primers used were as below:

TBP: 5’-TGTGCACAGGAGCCAAGAGT-3’ and 5’-ATTTTCTTGCTGCCAGTCTGG-3’;

NKX6.1: 5’-ATTCGTTGGGGATGACAGAG-3’ and 5’-CGAGTCCTGCTTCTTCTTGG-3’;

PDX1: 5’-CCCATGGATGAAGTCTACC-3’ and 5’-GTCCTCCTCCTTTTTCCAC-3’;

NGN3: 5’-GCTCATCGCTCTCTATTCTTTTGC-3’ and 5’-GGTTGAGGCGTCATCCTTTCT-3’;

INS: 5’-AAGAGGCCATCAAGCAGATCA-3’ and 5’-CAGGAGGCGCATCCACA-3’;

SOX17: 5’-GGCGCAGCAGAATCCAGA-3’ and 5’-CCACGACTTGCCCAGCAT-3’.

DNA methylation analysis

Methylation analysis of *OCT4* promoter was performed as described previously (Liu et al., 2014). Briefly, genomic DNA was extracted using TissueGen DNA Kit (CWBIO) and bisulphate converted with CpGenome Fast DNA Modification Kit (Millipore). The modified genomic DNA was then used as template to propagate specific *OCT4* promoter DNA segments mediated by primers: F, 5'-ATTTGTTTTTTGGGTAGTTAAAGGT-3'; R, 5'-CCAACTATCTTCATCTTAATAACATCC-3'. The DNA segments were sub-cloned into pMD19T vector using pMD-T Simple Vector kit (Takara), followed by sequencing.

Alkaline Phosphatase (AP) staining

AP staining was employed using Alkaline Phosphatase Staining Kit II (Stemgent). Briefly, the cells were fixed for 2 to 5 min with 4% formaldehyde, and incubated for 5 to 15 min with freshly prepared AP substrate solution at room temperature.

Immunofluorescence microscopy

Immunofluorescence staining was performed as previously described (Liu et al., 2012; Liu et al., 2014). Generally, cells were first fixed with 4% formaldehyde for 15-20 min and then permeabilized with 0.4% Triton X-100 for 15 min in PBS at room temperature, followed by blocked with 10% donkey serum in PBS for 1 hour. And after incubated with indicated primary antibody at 4 ℃ overnight, cells were incubated at room temperature with the corresponding secondary antibody and counterstained with Hoechst 33258 (Invitrogen) for 1 hour.

Embryonic Body (EB) formation *in vitro* and teratoma analysis *in vivo*

EB differentiation was performed as previously described with small modifications (Itskovitz-Eldor et al., 2000; Liu et al., 2011). Briefly, iPSC colonies cultured on plate with Matrigel coated were digested with dispase (Gibco) at 37℃ till about 80% colonies were dissociated. These colonies were then washed with DMEM/F12 twice and cultured on low attachment plate (Corning costar) in EB medium (85% IMDM (Gibco), 15% FBS (Gemini), 1% NEAA (Gibco), 1% L-GlutaMax (Gibco)) for about one week. In the next two weeks, the colonies were switched on gelatin-coated plate for spontaneous differentiation. The differentiation potential of iPSCs was also evaluated *in vivo* by teratoma formation assay. Briefly, NOD-SCID mice were anaesthetized and subcutaneously injected with indicated iPSCs. After 6-8 weeks, the teratoma formation was observed and the teratoma was isolated and assessed by immunofluorescence.

NSCs generation from iPSCs and neuronal differentiation assay

NSC and neuronal differentiation from iPSCs was performed as previously reported (Liu et al., 2012).

Pancreatic progenitor (PP) differentiation assay

PP differentiation was initiated till the confluence of iPSCs cultured on Matrigel-coated MEF feeder system reached to more than 80%. iPSCs were firstly cultured in RPMI 1640 (Gibco) containing 100 ng/ml activin A (R&D) for 4 days with 25 ng/ml Wnt3a (R&D) added for the first day to promote DE differentiation, followed by the induction with high glucose DMEM medium supplemented with 1% B27, 2 μM Retinoic acid (Sigma), 50 ng/ml FGF10 (R&D), 100 ng/ml Noggin (R&D) and 0.25 μM Cyclopamine-KAAD (Calbiochem-EMD Millipore) for 6 days. Furthermore, PP cells were induced by addition of DMEM high glucose medium containing 1% B27 and 50 ng/ml FGF10 (R&D) for 3 days.

References

Itskovitz-Eldor, J., Schuldiner, M., Karsenti, D., Eden, A., Yanuka, O., Amit, M., Soreq, H., and Benvenisty, N. (2000). Differentiation of human embryonic stem cells into embryoid bodies comprising the three embryonic germ layers. Mol Med 6, 88-95.

Liu, G.H., Barkho, B.Z., Ruiz, S., Diep, D., Qu, J., Yang, S.L., Panopoulos, A.D., Suzuki, K., Kurian, L., Walsh, C., et al. (2011). Recapitulation of premature ageing with iPSCs from Hutchinson-Gilford progeria syndrome. Nature 472, 221-225.

Liu, G.H., Qu, J., Suzuki, K., Nivet, E., Li, M., Montserrat, N., Yi, F., Xu, X., Ruiz, S., Zhang, W., et al. (2012). Progressive degeneration of human neural stem cells caused by pathogenic LRRK2. Nature 491, 603-607.

Liu, G.H., Suzuki, K., Li, M., Qu, J., Montserrat, N., Tarantino, C., Gu, Y., Yi, F., Xu, X., Zhang, W., et al. (2014). Modelling Fanconi anemia pathogenesis and therapeutics using integration-free patient-derived iPSCs. Nat Commun 5, 4330.

Okita, K., Matsumura, Y., Sato, Y., Okada, A., Morizane, A., Okamoto, S., Hong, H., Nakagawa, M., Tanabe, K., Tezuka, K., et al. (2011). A more efficient method to generate integration-free human iPS cells. Nature methods 8, 409-412.

Zhou, T., Benda, C., Dunzinger, S., Huang, Y., Ho, J.C., Yang, J., Wang, Y., Zhang, Y., Zhuang, Q., Li, Y., et al. (2012). Generation of human induced pluripotent stem cells from urine samples. Nat Protoc 7, 2080-2089.
